# Comparison of experimental data obtained using the *reference* and the single-serpentine proton exchange membrane single fuel cell testing hardware

**DOI:** 10.1016/j.dib.2020.105945

**Published:** 2020-06-27

**Authors:** Tomasz Bednarek, Georgios Tsotridis

**Affiliations:** European Commission, Joint Research Centre (JRC), Directorate C Energy Transport and Climate, The Netherlands

**Keywords:** Single pem fuel cell, Reference testing hardware, Single serpentine fuel cell, Temperature, Pressure and voltage distributions, Polarisation curve, Electrochemical impedance spectroscopy

## Abstract

The paper presents testing data of single Polymer Electrolyte Membrane (PEM) fuel cell for comparing the characteristics of two different testing hardware, namely the recently developed single cell *reference* testing hardware and the commonly used by the research community single-serpentine testing hardware. Data are presented in form of polarisation curves, temperature, pressure and voltage distributions, as well as electrochemical impedance spectroscopy measurements for a range of current densities. The electrochemical impedance spectroscopy data are also validated using the Kramers-Kronig transformation and presented as Nyquist and Bode plots. The raw data of all mentioned results are also provided in online repository. The presented experimental data are discussed and evaluated in *Assessment of the electrochemical characteristic of a Polymer Electrolyte Membrane in a “reference” single fuel cell testing hardware*[1].

Specifications table

**Table:** 

Subject	Energy Engineering and Power Technology
Specific subject area	Electrochemical characterisation of PEM fuel cell MEAs using single cell testing hardware
Type of data	Table Plot Raw data
How data were acquired	The Polymer Electrolyte Membrane (PEM) single fuel cell experiments were performed at the GreenLight 611–620 testing station. Two different single cell testing hardware were used: the newly developed single cell testing hardware called “JRC ZERO∇CELL” of 10cm^2^ active area (20 × 50 mm length) and the commonly used by the research community single-serpentine hardware with 25 cm^2^ active area (50 × 50 mm).
Data format	Plots Analysed Raw data available through online repository
Parameters for data collection	The operating conditions for the execution of the experiments were modified form the *EU Harmonised Test Protocols for PEMFC MEA Testing in Single Cell Configuration for Automotive Applications*[2].
Description of data collection	Data were collected from single fuel cell experiments performed at the two different testing hardware by monitoring the distributions of temperature, pressure cell voltage, as well as by performing polarisation curve measurements and electrochemical impedance spectroscopy analysis in galvanostatic mode for a range of current densities.
Data source location	European Commission, Joint Research Centre 1755 LE Petten Westerduinweg 3 The Netherlands
Data accessibility	Direct URL to data: https://data.mendeley.com/datasets/n5csdjfg3c/draft?a=fcdaa6b0-4a23-4fc2-91a1-55eabeb3245f doi: 10.17632/n5csdjfg3c.2
Related research article	[1] Tomasz Bednarek & Georgios Tsotridis, *Assessment of the electrochemical characteristic of a Polymer Electrolyte Membrane in a “reference” single fuel cell testing hardware*, Journal of Power Sources, in press, https://doi.org/10.1016/j.jpowsour.2020.228319.

## Value of the data

• The presented data are useful because they demonstrate the comparison of the testing characteristics of two different testing hardware setups, namely the one developed by the Joint Research Centre and hereafter called JRC ZERO∇CELL [1] and the single-serpentine cell.
• The data will benefit researchers for MEA development for PEM fuel cells development, since the new testing hardware provides an improved single cell testing platform by providing as uniform as possible testing conditions, hence improving the accuracy of assessment of the electrochemical characteristics of the MEAs.
• The testing data can be further used to support future developments of PEM fuel cell materials.
• The additional value of the obtained data, demonstrates the high quality of experimental data obtained using the JRC ZERO∇CELL in terms of accuracy, stability and repeatability, hence suggesting that this hardware, could be adopted by the research community as the *reference* PEM single cell testing hardware. To this respect, it will provide the opportunity to compare research results published in the literature, hence to accelerate the development and deployment of PEM fuel cell technology.

## 1 Data description

The current study presents raw experimental data logs as well as polarisation curves (IV curve) and Electro-chemical Impedance Spectroscopy (EIS) measurements. The experiments were performed using the same GreenLight G11-620 test station for both investigated single-cell testing setups, namely: the JRC ZERO∇CELL and Single-Serpentine HardWare (S-S HW). The data are logged with frequency 1 Hz.

The raw experimental data presented in this paper are available in on-line repository [3]:

v The file ‘S-S_HW_IV_EIS.xlsx’ contains experimental data obtained using the S-S HW.
v While the file ‘ZEROCELL_IV_EIS.xlsx’ contains data obtained using the JRC ZERO∇CELL.

Both data files keep the same data structure. The exact location of the data used in presented plots is explained in the following sections.

## 2 Experimental design, materials and methods

The JRC ZERO∇CELL single-cell testing hardware is characterised by 10 cm^2^ active area, 20 mm wide and 50 mm long, with parallel channels flow field pattern. All features of the JRC ZERO∇CELL are provided in [1].

The second testing setup used in this study, the S-S HW, is commonly employed by the research community for single PEM fuel cell performance and durability tests. It has an active area of 25 cm^2^ (50 × 50 mm) and is provided by balticFuelCells GmbH.

The tested Membrane Electro Assemblies (MEAs) for JRC ZERO∇CELL and S-S HW are composed of Catalyst Coated Membrane (CCM) laminated in mylar frame in order to reinforce membrane edges, increase mechanical strength as well as to precisely limit the active area size. The Gas Diffusion Layer (GDL) is placed on both sides of this mylar-CMM laminate. The CCM specimens and the GDL layers are taken from the same batch of materials to avoid discrepancies between the MEAs examined using the two testing set-ups. The properties of the examined MEAs are listed in Table 1, and are also presented in [1].

**Table 1:** Specification of the examined MEAs, [1].

	MEA
Active area size	10 cm^2^, 20 × 50 mm (JRC ZERO∇CELL) 25 cm^2^, 50 × 50 mm (S-S HW)
Membrane thickness	∼20 μm
Anode catalyst	Pt, loading 0.15÷0.25 mg cm^−2^
Cathode catalyst	Pt, loading 0.35÷0.45 mg cm^−2^
Anode GDL	Sigracet GDL 25BC
Cathode GDL	Sigracet GDL 25BC
Sub-gasket material	Mylar
Sealing gasket material	Viton

### 2.1 Polarisation curve experiments

The polarisation curve experiments were performed according to the protocol adopted from [4] in stoichiometric and galvanostatic modes, starting from open-circuit conditions up to maximum possible achievable current density at voltage of ∼0.25 V (forward current density sweep), and repeated again in reverse order, from the maximum current density back to open-circuit condition (backward current density sweep). The applied operating conditions were discussed in [1] and are presented in Table 2.

**Table 2:** Operating conditions for S-S HW and for JRC ZERO∇CELL [1].

Parameters		Operating conditions		
		Unit	S-S HW	JRC ZERO∇CELL
	C ell operating temperature	°C	80	80
ANODE	F uel gas inlet temperature	°C	85	85
	Fuel gas inlet humidity*	%	100	100
	Fuel gas inlet pressure (absolute)	kPa	250	250
	Fuel inlet stoichiometry ratio	–	1.3	8.0

CATHODE	O xidant gas inlet temperature	°C	85	85
	Oxidant gas inlet humidity*	%	100	100
	Oxidant pressure inlet (absolute)	kPa	230	230
	Oxidant inlet stoichiometry ratio	–	1.5	10.0

⁎ Inlet gas relative humidity at the cell operating temperature.

**Fig. 1:**
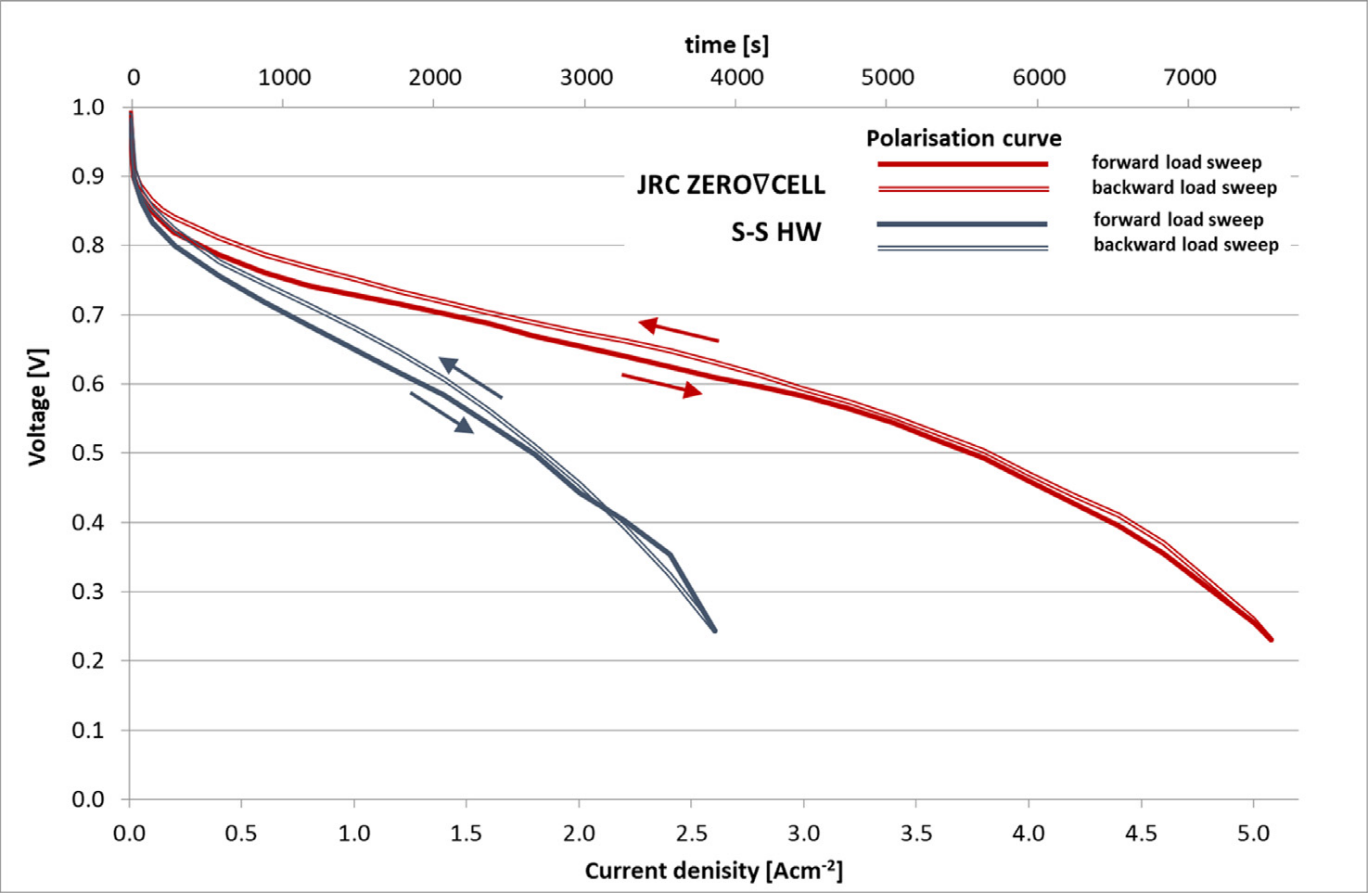
Polarisation curves and their slopes obtained using the JRC ZERO∇CELL and the S-S HW, MEA Pt loading: anode 0.15÷0.25 mg cm^−2^, cathode 0.35÷0.45 mg cm^−2^.

The polarisation curves using the JRC ZERO∇CELL and S-S HW are presented in Fig. 1. The analysis of the examined results are presented at length in [1]. The plots presented in Fig. 1 can be reproduced from the data provided in [3], sheet ‘data’, columns AM (current density) and AN (cell voltage) in files ‘ZEROCELL_HW_IV_EIS.xlsx’ and ‘S-S_HW_IV_EIS.xlsx’ for experiments performed using the JRC ZERO∇CELL and S-S HW respectively.

### 2.2 Voltage evolution

**Fig. 2:**
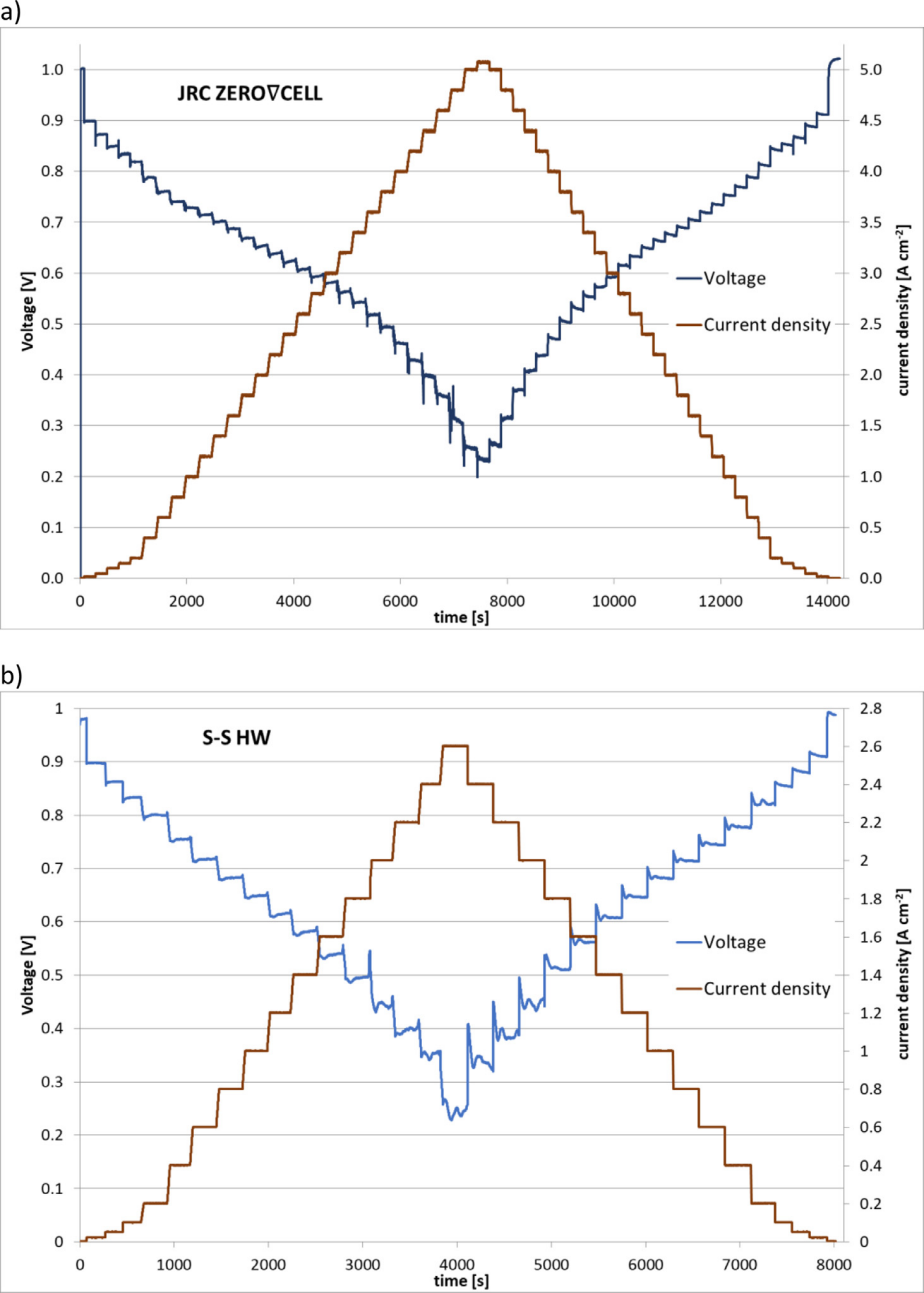
Voltage development during polarisation curve experiment using: a) the JRC ZERO∇CELL and b) the S-S HW.

**Fig. 3:**
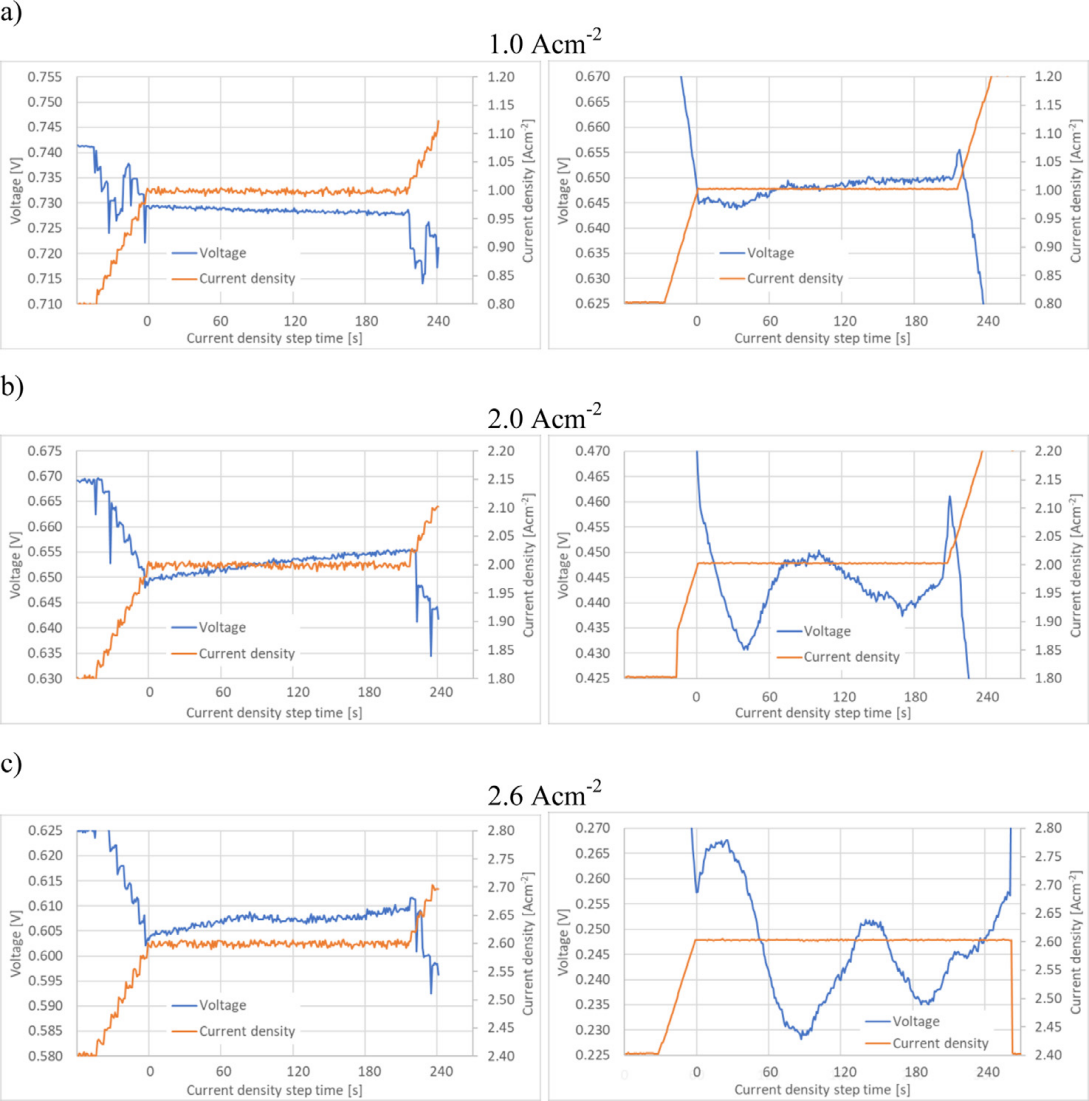

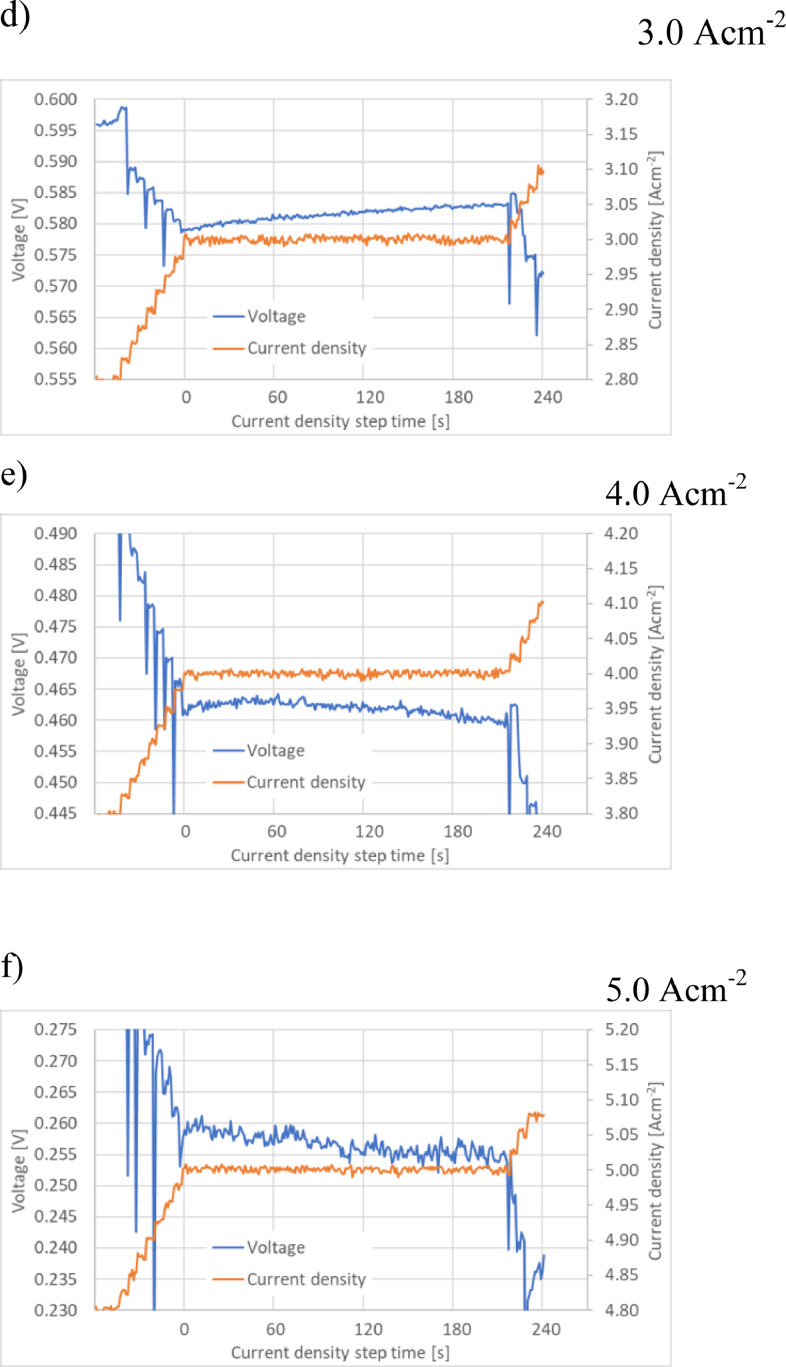
Voltage distribution during polarization curve experiments performed using the JRC ZERO∇CELL at current density steps: 3.1) 1.0  Acm^−2^, 3.3) 2.0 Acm^−2^, 3.5) 2.6 Acm^−2^ (maximum current density for S-S HW), 3.7) 3.0 Acm^−2^, 3.8) 4.0 Acm^−2^, 3.9) 5.0 Acm^−2^ (maximum current density for the JRC ZERO∇CELL) and for S-S HW at current density steps: 3.2) 1.0 Acm^-2^, 3.4) 2.0 Acm^−2^ and 3.6) 2.6 Acm^−2^ (maximum current density for S-S HW).

The evolution of voltage at different current density steps during the polarisation curve measurements, as well the “expanded” voltage fluctuations at several current density levels, for both JRC ZERO∇CELL and S-S HW testing hardware are presented in Fig. 2, Fig. 3. The cell operation at each current density step was hold for 180 s to ensure steady conditions and then the voltage was averaged over 30 s. The fluctuations (spikes) observed in Fig. 3, at the beginning and at the end of each current density step are associated with current changes due to the load bank of the test bench. For this reason, these artefacts are disregarded from the evaluation of the experimental data.

The raw experimental data used in Fig. 2 and Fig. 3 are available in [3]. Data files ‘ZEROCELL_HW_IV_EIS.xlsx’ and ‘S-S_HW_IV_EIS.xlsx’ correspond to experiments performed using the JRC ZERO∇CELL and S-S HW respectively. The data are located in sheet ‘data’, columns B (current density) and C (cell voltage).

### 2.3 Pressure distribution

**Fig. 4:**
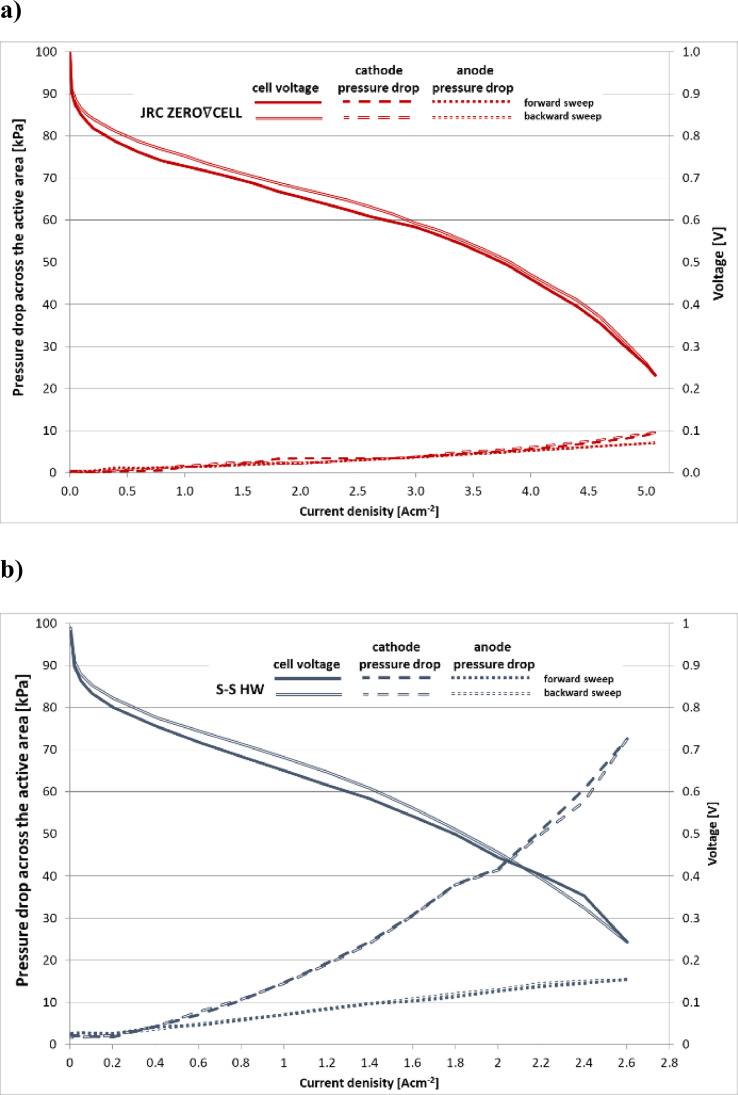
The values of pressure drop across the active area in anode and cathode compartments during polarisation curve experiment for: a) the JRC ZERO∇CELL and b) S-S HW.

**Fig. 5:**
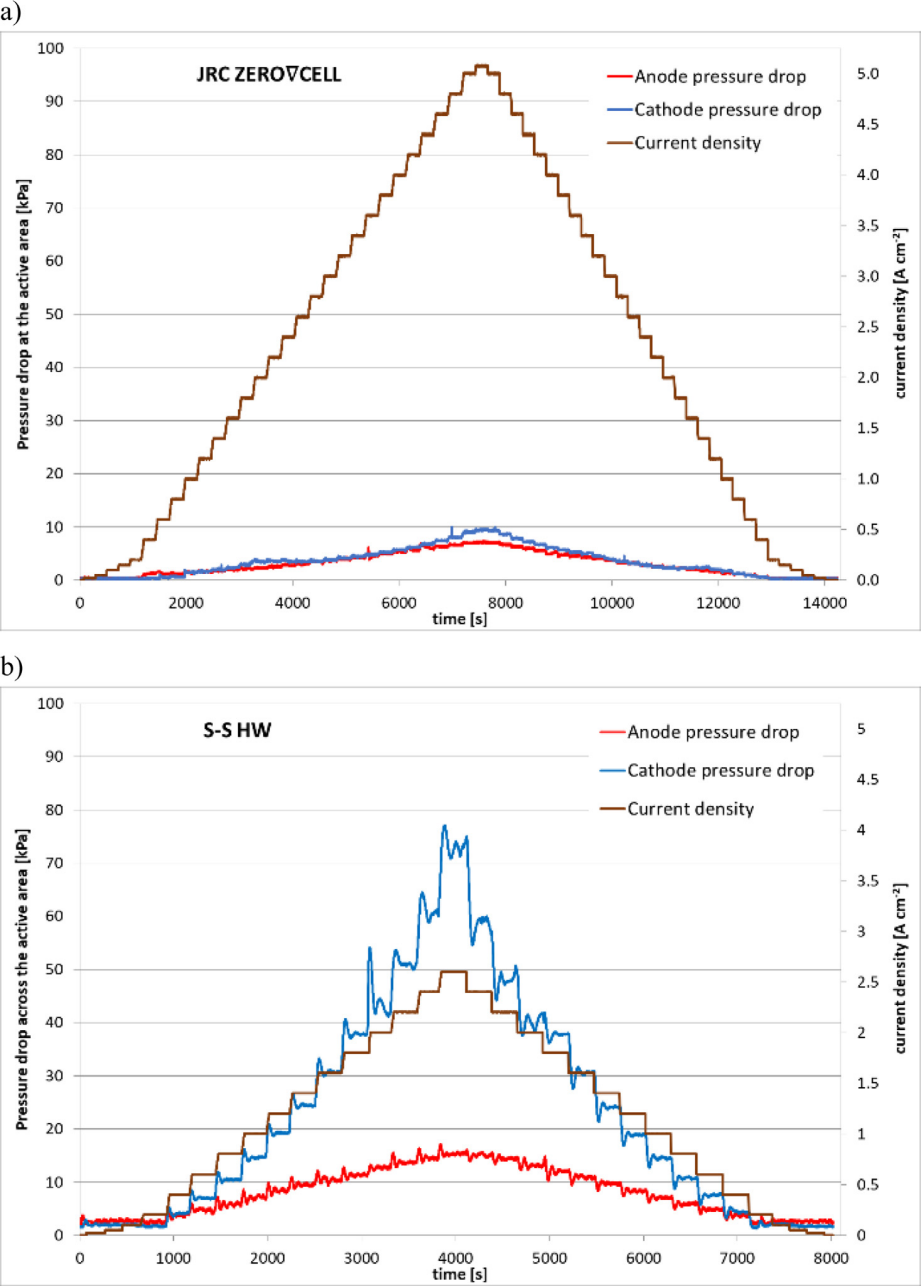
Evolution in time of pressure drop of reactant gasses across the active area for: a) the JRC ZERO∇CELL and S-S HW.

The plots of averaged pressure drop across the active area over 30 s of steady operation of the two investigated testing setups are presented in Fig. 4, whereas, the evolution of pressure drop fluctuations during the polarisation test experiments for both testing setups is presented in Fig. 5.

The plots presented in Fig. 4 and Fig. 5 were based on the raw data provided in [3], files ‘ZEROCELL_HW_IV_EIS.xlsx’ and ‘S-S_HW_IV_EIS.xlsx’ correspond to experiments performed using the JRC ZERO∇CELL and S-S HW respectively. The averaged values of pressure drop at consecutive current density steps are located in sheet ‘data’, columns AN (current density), AQ (pressure drop in anode compartment) and AS (pressure drop in cathode compartment), whereas the values of pressure drop logged by the test station during the polarisation curve experiment for both anode and cathode compartments are located in columns K and H respectively.

### 2.4 Temperature distribution

The JRC ZERO∇CELL testing hardware provides the possibility of temperature monitoring at both anode and cathode compartments, whereas S-S HW has only one thermocouple at the cathode bi-polar plate. Therefore, the evaluation of spatial temperature distribution is possible only for experiments performed using the JRC ZERO∇CELL.

**Fig. 6:**
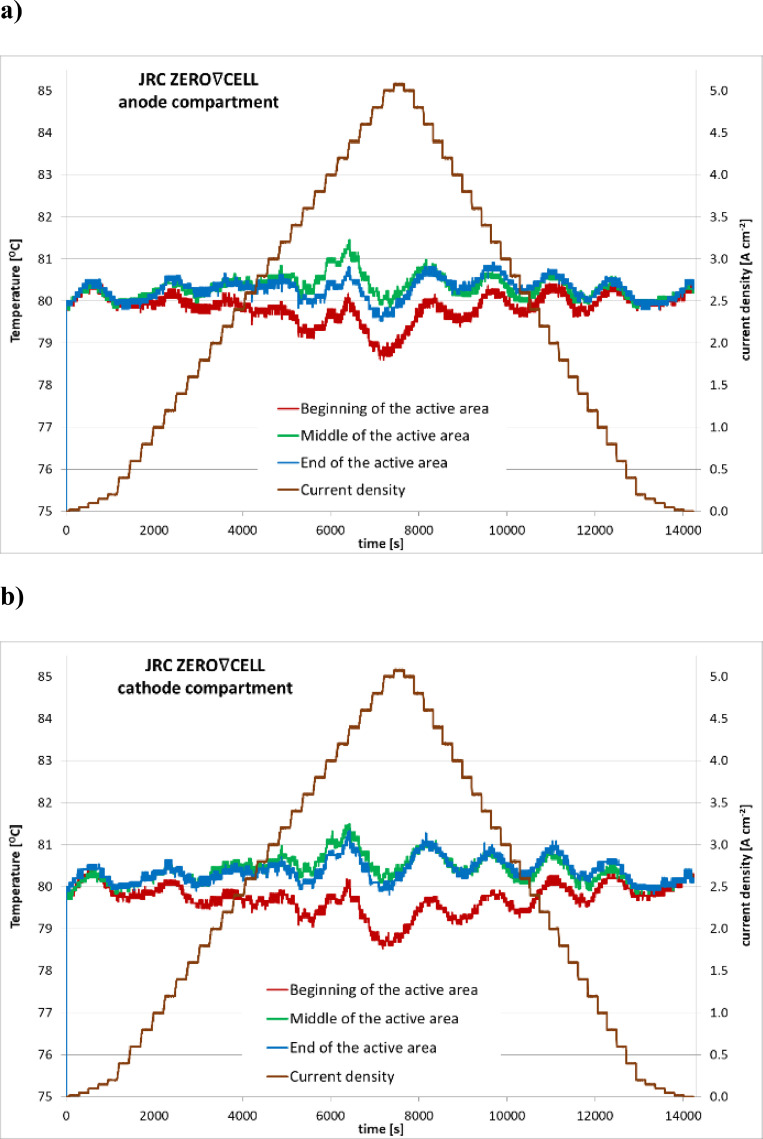
Time evolution of temperature in anode(a) and cathode (b) compartments.

The time evolution of temperature at the beginning, middle and end of the active area at both compartments, anode and cathode, during the polarisation curve measurements using the JRC ZERO∇CELL is presented in Fig. 6. The raw experimental data are available in [3], file ‘ZEROCELL_HW_IV_EIS.xlsx’, sheet ‘data’. The temperature logs for anode compartment are located in columns P, Q and R (beginning, middle and end of the active area respectively) while temperature recorded in cathode compartment is located in columns Z, AA and AB.

### 2.5 Electro-chemical impedance spectroscopy measurements

Electro-chemical Impedance Spectroscopy tests [5,6] were performed at operating conditions given in Table 2 in galvanostatic mode at a range of operating current densities, from 0.5Acm^−2^ until the cell voltage is greater than 0.4 V. Each measurement is preceded by 10 min steady operation to achieve steady-state conditions in terms of temperature, membrane hydration and liquid water distribution in GDL and gas channels.

The amplitude of current perturbation was adjusted to the obtained voltage response equal to ±10 mV at 1 Hz. The impedance measurements were performed from 10 kHz down to 0.5 Hz (6 points per decade of frequency) and from 10 kHz down to 1 Hz (10 points per decade of frequency) using JRC ZERO∇CELL and S-S HW respectively.

**Fig. 7:**
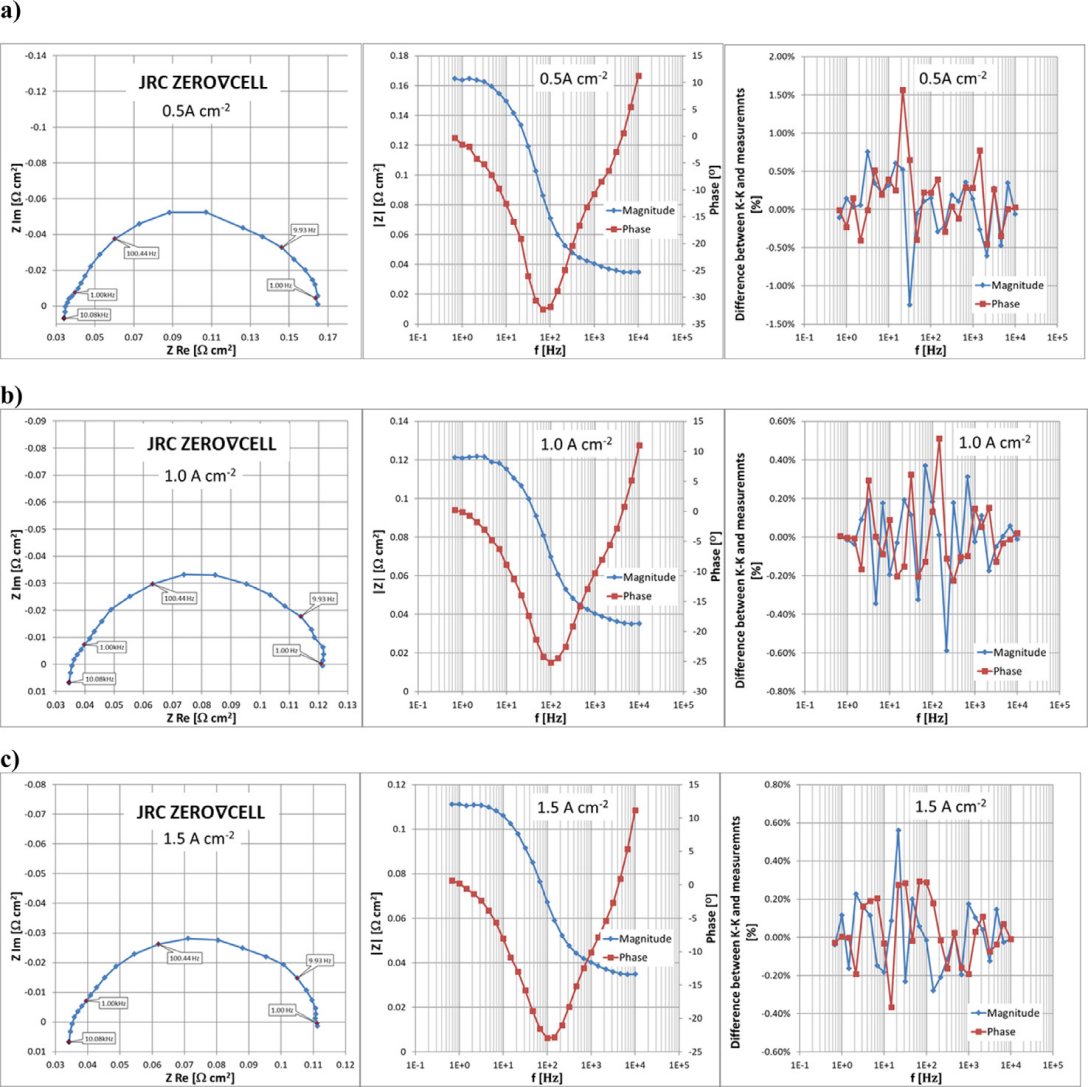

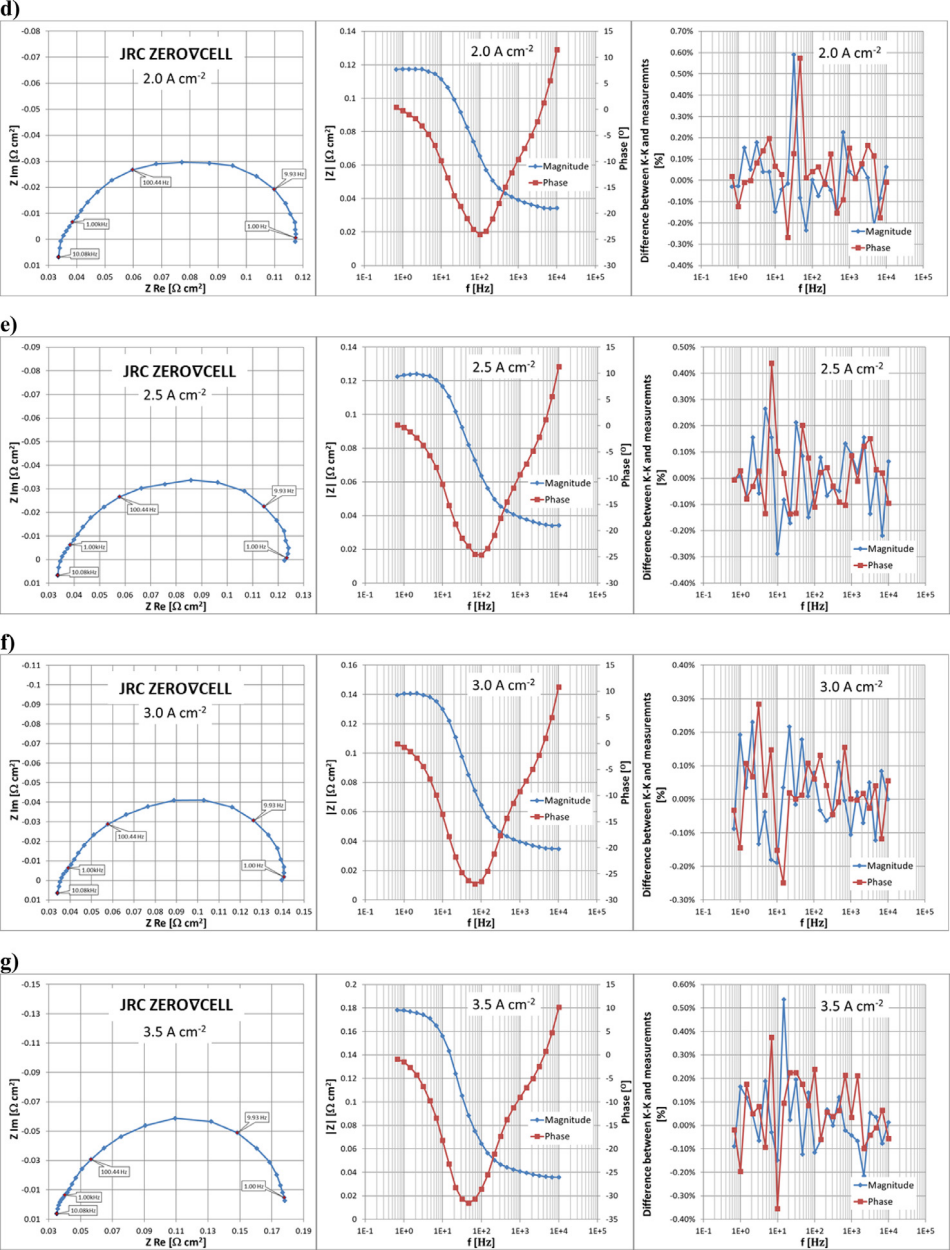
Results of EIS measurements using JRC ZERO∇CELL at a range of operating current densities from 0.5 to 3.5 Acm^−2^ and validation of measurements using the Kramers–Kronig transform.

**Fig. 8:**
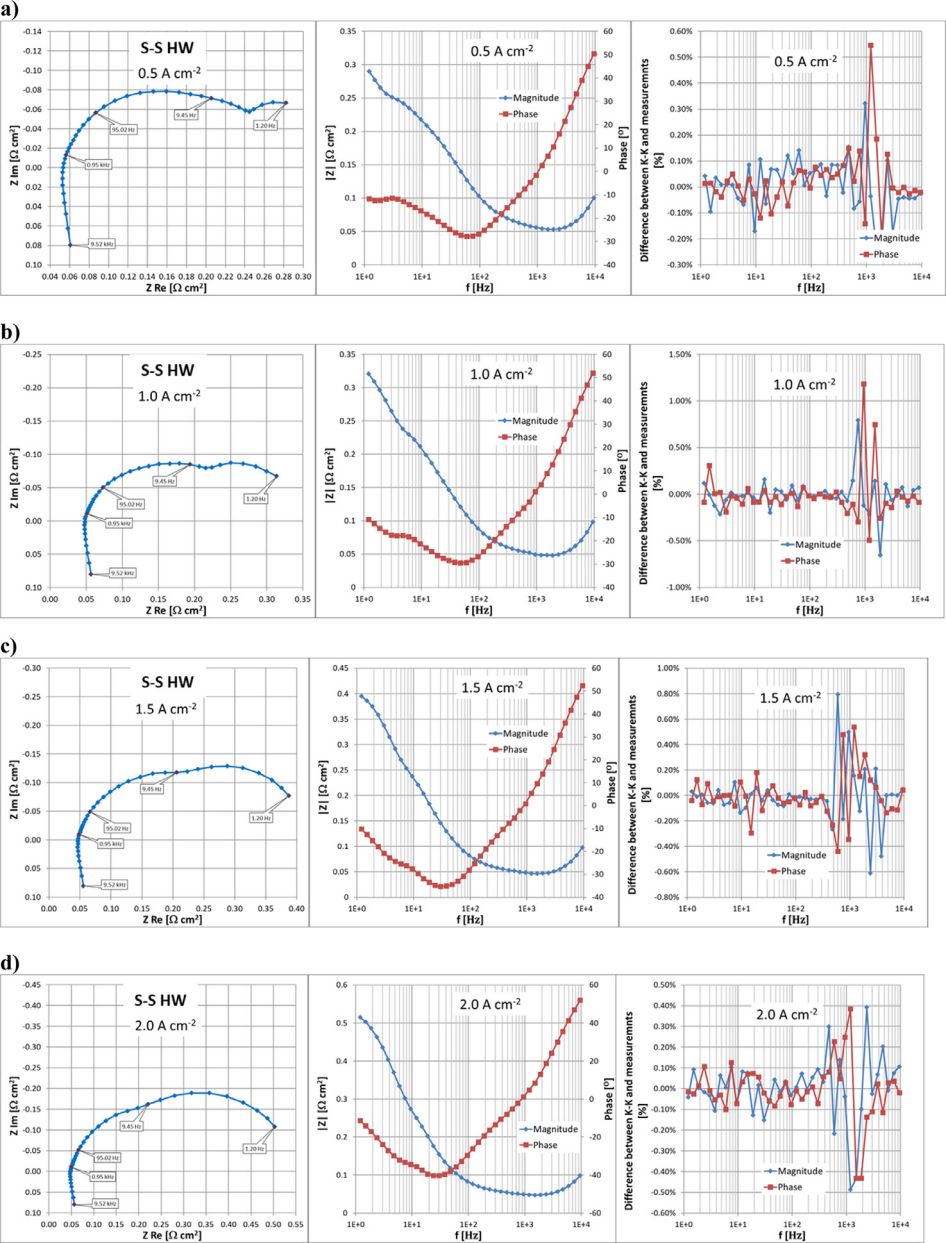
Results of EIS measurements using S-S HW at a range of operating current densities from 0.5 to 2.0 Acm^−2^ and validation of measurements using the Kramers–Kronig transform.

The results of EIS measurements are presented in Fig. 7, Fig. 8 respectively for the JRC ZERO∇CELL and S-S HW respectively as Nyquist (real vs imaginary impedance) and Bode (impedance magnitude and phase angle vs perturbation frequency) plots. The stability and linear regime of EIS measurements are validated using Kramers-Kronig (K-K) transformation [7].

The resulting data on EIS measurements are available in [3]. Sheet ‘EIS_data’ in files ‘ZEROCELL_HW_IV_EIS.xlsx’ and ‘S-S_HW_IV_EIS.xlsx’ contains full information on the performed EIS measurements using the JRC ZERO∇CELL and S-S HW respectively. The real and imaginary parts of the obtained impedance values are located in columns P and Q respectively, while impedance magnitude is located in column R and phase angle is given in column I. The relative difference (in percentage) between the measured impedance values and K-K transformation for impedance magnitude and phase are given in columns S and T respectively,

## Declaration of Competing Interest

The authors declare that they have no known competing financial interests or personal relationships which have, or could be perceived to have, influenced the work reported in this article.
